# Public Health Assessment of Potential Biological Terrorism Agents

**DOI:** 10.3201/eid0802.010164

**Published:** 2002-02

**Authors:** Lisa D. Rotz, Ali S. Khan, Scott R. Lillibridge, Stephen M. Ostroff, James M. Hughes

**Affiliations:** Centers for Disease Control and Prevention, Atlanta, Georgia, USA

**Keywords:** bioterrorism, biological agents, bioterrorism preparedness

As part of a Congressional initiative begun in 1999 to upgrade national public health capabilities for response to acts of biological terrorism, the Centers for Disease Control and Prevention (CDC) was designated the lead agency for overall public health planning. A Bioterrorism Preparedness and Response Office has been formed to help target several areas for initial preparedness activities, including planning, improved surveillance and epidemiologic capabilities, rapid laboratory diagnostics, enhanced communications, and medical therapeutics stockpiling (*1*). To focus these preparedness efforts, however, the biological agents towards which the efforts should be targeted had to first be formally identified and placed in priority order. Many biological agents can cause illness in humans, but not all are capable of affecting public health and medical infrastructures on a large scale.

The military has formally assessed multiple agents for their strategic usefulness on the battlefield (*2*). In addition, the Working Group on Civilian Biodefense, using an expert panel consensus-based process, has identified several biological agents as potential high-impact agents against civilian populations (*3*–*7*). To guide national public health bioterrorism preparedness and response efforts, a method was sought for assessing potential biological threat agents that would provide a reviewable, reproducible means for standardized evaluations of these threats.

In June 1999, a meeting of national experts was convened to 1) review potential general criteria for selecting the biological agents that pose the greatest threats to civilians and 2) review lists of previously identified biological threat agents and apply these criteria to identify which should be evaluated further and prioritized for public health preparedness efforts. This report outlines the overall selection and prioritization process used to determine the biological agents for public health preparedness activities. Identifying these priority agents will help facilitate coordinated planning efforts among federal agencies, state and local emergency response and public health agencies, and the medical community.

## Overview of Agent Selection and Prioritization Process

On June 3-4, 1999, academic infectious disease experts, national public health experts, Department of Health and Human Services agency representatives, civilian and military intelligence experts, and law enforcement officials (see footnote) met to review and comment on the threat potential of various agents to civilian populations. The following general areas were used as criteria: 1) public health impact based on illness and death; 2) delivery potential to large populations based on stability of the agent, ability to mass produce and distribute a virulent agent, and potential for person-to-person transmission of the agent; 3) public perception as related to public fear and potential civil disruption; and 4) special public health preparedness needs based on stockpile requirements, enhanced surveillance, or diagnostic needs. Participants reviewed lists of biological warfare or potential biological threat agents and selected those they felt posed the greatest threat to civilian populations.

The following unclassified documents containing potential biological threat agents were reviewed: 1) the Select Agent Rule list, 2) the Australian Group List for Biological Agents for Export Control, 3) the unclassified military list of biological warfare agents, 4) the Biological Weapons Convention list, and 5) the World Health Organization Biological Weapons list (*8*–*12*). Participants with appropriate clearance levels reviewed intelligence information regarding classified suspected biological agent threats to civilian populations. Genetically engineered or recombinant biological agents were considered but not included for final prioritization because of the inability to predict the nature of these agents and thus identify specific preparedness activities for public health and medical response to them. In addition, no information was available about the likelihood for use of one biological agent over another. This aspect, therefore, could not be considered in the final evaluation of the potential biological threat agents.

Participants discussed and identified agents they felt had the potential for high impact based on subjective assessments in the four general categories. After the meeting, CDC personnel then attempted to identify objective indicators in each category that could be used to further define and prioritize the identified high-impact agents and provide a framework for an objective risk-matrix analysis process for any potential agent. The agents were evaluated in each of the general areas according to the objective parameters and were characterized by the rating schemes outlined in the Appendix. Final category assignments (A, B, or C) of agents for public health preparedness efforts were then based on an overall evaluation of the ratings the agents received in each of the four areas.

## Results

Based on the overall criteria and weighting, agents were placed in one of three priority categories for initial public health preparedness efforts: A, B, or C (Table 1). Agents in Category A have the greatest potential for adverse public health impact with mass casualties, and most require broad-based public health preparedness efforts (e.g., improved surveillance and laboratory diagnosis and stockpiling of specific medications). Category A agents also have a moderate to high potential for large-scale dissemination or a heightened general public awareness that could cause mass public fear and civil disruption.

**Table 1:** Critical biological agent categories for public health preparedness

Biological agent(s)	Disease
**Category A**	
*Variola major*	Smallpox
*Bacillus anthracis*	Anthrax
*Yersinia pestis*	Plague
*Clostridium botulinum* (botulinum toxins)	Botulism
*Francisella tularensis*	Tularemia
Filoviruses and Arenaviruses (e.g.*, Ebola virus, Lassa virus*)	Viral hemorrhagic fevers

**Category B**	
*Coxiella burnetii*	Q fever
*Brucella spp.*	Brucellosis
*Burkholderia mallei*	Glanders
*Burkholderia pseudomallei*	Melioidosis
*Alphaviruses* (VEE, EEE, WEE^a^)	Encephalitis
*Rickettsia prowazekii*	Typhus fever
Toxins (e.g., Ricin, Staphylococcal enterotoxin B)	Toxic syndromes
*Chlamydia psittaci*	Psittacosis
Food safety threats (e.g., *Salmonella spp*., *Escherichia coli* O157:H7)	
Water safety threats (e.g., *Vibrio cholerae*, Cryptosporidium parvum)	

**Category C**
Emerging threat agents (e.g., *Nipah virus*, hantavirus)

^a^Venezuelan equine (VEE), eastern equine (EEE), and western equine encephalomyelitis (WEE) viruses

Most Category B agents also have some potential for large-scale dissemination with resultant illness, but generally cause less illness and death and therefore would be expected to have lower medical and public health impact. These agents also have lower general public awareness than Category A agents and require fewer special public health preparedness efforts. Agents in this category require some improvement in public health and medical awareness, surveillance, or laboratory diagnostic capabilities, but presented limited additional requirements for stockpiled therapeutics beyond those identified for Category A agents. Biological agents that have undergone some development for widespread dissemination but do not otherwise meet the criteria for Category A, as well as several biological agents of concern for food and water safety, are included in this category.

Biological agents that are currently not believed to present a high bioterrorism risk to public health but which could emerge as future threats (as scientific understanding of these agents improves) were placed in Category C. These agents will be addressed nonspecifically through overall bioterrorism preparedness efforts to improve the detection of unexplained illnesses and ongoing public health infrastructure development for detecting and addressing emerging infectious diseases (*13*).

Agents were categorized based on the overall evaluation of the different areas considered. Table 2 shows the evaluation schemes as applied to agents in Categories A and B. For example, smallpox would rank higher than brucellosis in the public health impact criterion because of its higher untreated mortality (approximately 30% for smallpox and ≤2% for brucellosis); smallpox has a higher dissemination potential because of its capability for person-to-person transmission. Smallpox also ranks higher for special public health preparedness needs, as additional vaccine must be manufactured and enhanced surveillance, educational, and diagnostic efforts must be undertaken. Inhalational anthrax and plague also have higher public health impact ratings than brucellosis because of their higher morbidity and mortality. Although mass production of *Vibrio cholera* (the biological cause of cholera) and *Shigella* spp. (the cause of shigellosis) would be easier than the mass production of anthrax spores, the public health impact of widespread dissemination would be less because of the lower morbidity and mortality associated with these agents. Although the infectious doses of these bacteria are generally low, the total amount of bacteria that would be required and current water purification and food-processing methods would limit the effectiveness of intentional large-scale water or food contamination with these agents.

**Table 2:** Criteria and weighting^a^ used to evaluate potential biological threat agents

Disease	Public health impact		Dissemination potential		Public perception	Special preparation	Category
	Disease	Death	P-D^b^	P - P^c^			
Smallpox	+	++	+	+++	+++	+++	A
Anthrax	++	+++	+++	0	+++	+++	A
Plague^d^	++	+++	++	++	++	+++	A
Botulism	++	+++	++	0	++	+++	A
Tularemia	++	++	++	0	+	+++	A
VHF^e^	++	+++	+	+	+++	++	A
VE^f^	++	+	+	0	++	++	B
Q Fever	+	+	++	0	+	++	B
Brucellosis	+	+	++	0	+	++	B
Glanders	++	+++	++	0	0	++	B
Melioidosis	+	+	++	0	0	++	B
Psittacosis	+	+	++	0	0	+	B
Ricin toxin	++	++	++	0	0	++	B
Typhus	+	+	++	0	0	+	B
Cholera^g^	+	+	++	+/-	+++	+	B
Shigellosis^g^	+	+	++	+	+	+	B

^a^Agents were ranked from highest threat (+++) to lowest (0). ^b^Potential for production and dissemination in quantities that would affect a large population, based on availability, BSL requirements, most effective route of infection, and environmental stability. ^c^Person-to-person transmissibility. ^d^Pneumonic plague. ^e^Viral hemorrhagic fevers due to Filoviruses (*Ebola, Marburg*) or Arenaviruses (e.g., *Lassa, Machupo*). ^f^Viral encephalitis. ^g^Examples of food- and waterborne diseases.

## Discussion

Although use of conventional weapons such as explosives or firearms is still considered the most likely means by which terrorists could harm civilians (*14*), multiple recent reports cite an increasing risk and probability for the use of biological or chemical weapons (*15*–*18*). Indeed, the use of biological and chemical agents as small- and large-scale weapons has been actively explored by many nations and terrorist groups (*19*,*20*). Although small-scale bioterrorism events may actually be more likely in light of the lesser degrees of complexity to be overcome, public health agencies must prepare for the still-possible large-scale incident that would undoubtedly lead to catastrophic public health consequences. The selection and prioritization of the potential biological terrorism agents described in this report were not based on the likelihood of their use, but on the probability that their use would result in an overwhelming adverse impact on public health.

Most evaluations of potential risk agents for biological warfare or terrorism have historically been based on military concerns and criteria for troop protection. However, several characteristics of civilian populations differ from those of military populations, including a wider range of age groups and health conditions, so that lists of military biological threats cannot simply be adopted for civilian use. These differences and others may greatly increase the consequences of a biological attack on a civilian population. Civilians may also be more vulnerable to food- or waterborne terrorism, as was seen in the intentional *Salmonella* contamination of salad bars in The Dalles, Oregon, in 1984 (*21*). Although food and water systems in the United States are among the safest in the world, the occurrence of nationwide outbreaks due to unintentional food or water contamination demonstrates the ongoing need for vigilance in protecting food and water supplies (*22*,*23*). Overall, many other factors must be considered in defining and focusing multiagency efforts to protect civilian populations against bioterrorism.

Category A agents are being given the highest priority for preparedness. For Category B, public health preparedness efforts will focus on identified deficiencies, such as improving awareness and enhancing surveillance or laboratory diagnostic capabilities. Category C agents will be further assessed for their potential to threaten large populations as additional information becomes available on the epidemiology and pathogenicity of these agents. In addition, special epidemiologic and laboratory surge capacity will be maintained to assist in the investigation of naturally occurring outbreaks due to Category C “emerging” agents. Linkages established with established programs for food safety, emerging infections diseases, and unexplained illnesses will augment the overall bioterrorism preparedness efforts for many Category B and C agents.

The above categories of agents should not be considered definitive. The prioritization of biological agents for preparedness efforts should continue. Agents in each category may change as new information is obtained or new assessment methods are established. Disease elimination and eradication efforts may result in new agents being added to the list as populations lose their natural or vaccine-induced immunity to these agents. Conversely, the priority status of certain agents may be reduced as the identified public health and medical deficiencies related to these agents are addressed (e.g., once adequate stores of smallpox vaccine and improved diagnostic capabilities are established, its rating within the special preparedness needs category would be reduced, as would its overall rating within the risk-matrix evaluation process). To meet the ever-changing response and preparedness challenges presented by bioterrorism, a standardized and reproducible evaluation process similar to the one outlined above will continue to be used to evaluate and prioritize currently identified biological critical agents, as well as new agents that may emerge as threats to civilian populations or national security.

## Supplementary Material

Appendix Risk-Matrix Analysis Process Used to Evaluate Potential Biological Threat Agents
