# Supplementary material for: Public Health Assessment of Potential Biological Terrorism Agents
Source: Emerg Infect Dis. 2002 Feb;8(2):225–30. doi: 10.3201/eid0802.010164 (PMC2732458; doi:10.3201/eid0802.010164)
Supplement: Appendix — Risk-Matrix Analysis Process Used to Evaluate Potential Biological Threat Agents [file 01-0164_app-s1.pdf]

Publisher: CDC; Journal: Emerging Infectious Diseases
Article Type: Online Conference Summary; Volume: 08; Issue: 02; Year: 2002; Article ID: 01-0164
DOI: 10.321/eid0802.010164; TOC Head: Online Conference Summary

Appendix

Risk-Matrix Analysis Process Used to Evaluate Potential Biological Threat Agents

In the area of public health impact, disease threat presented by an agent was assessed by evaluating whether the illness resulting from exposure could be treated without hospitalization. In addition, mortality rates for exposed, untreated persons were considered (1–3). Biological agents were given a higher rating for morbidity (++) if illness would most likely require hospitalization and a lower rating (+) if outpatient treatment might be possible for a large part of the affected population. Agents were also rated highest (+++) for expected untreated mortality ≥50%, medium (++) for mortality of 21% to 49%, and lowest (+) for an expected mortality ≤20%.

Agents were rated according to their overall potential for initial dissemination to a large population (+ to +++) and their potential for continued propagation by person-to-person transmission (0 to ++). Overall dissemination potential of an agent was based on an assessment of 1) the capability for mass production of the agent (assessment based on availability of agent and Biosafety Level (BSL) requirements for quantity production of an agent), and 2) their potential for rapid, large-scale dissemination (assessment based on the most effective route of infection and the general environmental stability of the agent). Agents were rated (++) if they were readily obtainable from soil, animal/insect, or plant sources (most available; e.g., *B. anthracis*), (+) if mainly available only from clinical specimens, clinical laboratories, or regulated commercial culture suppliers (e.g., *Shigella* spp.), and (0) if available only from nonenvironmental, noncommercial, or nonclinical sources such as high-level security research laboratories (least readily available; e.g., *Variola* or *Ebola* viruses).

BSL requirements for an agent were based on recommended levels for working with large quantities of an agent (4). BSL ratings were used to estimate the level of technical expertise and containment facilities that would be required to work with and mass produce an agent safely. Agents that required higher BSL levels were given lower ratings, as they would require greater

technical capabilities and containment facilities to be produced in large quantities. Agents were given (+) for BSL 4 production safety requirements, (++) for BSL 3 requirements, and (+++) for BSL 2 or lower requirements.

Agents were also assessed with regard to their main routes of infection, with the assumption that those causing infection via the respiratory route could be more readily disseminated to affect large populations. Agents were assigned (++) if most effective at causing illness via an aerosol exposure route (air release potential) and (+) if most effective when given by the oral route (food/water release potential). Dissemination potential should also take into account the stability of an agent following its release. Information regarding the expected general environmental stability of agents was obtained from multiple sources (1,5–8). Agents that may remain viable in the environment for ≥1 year were given (+++), while agents considered less environmentally stable were given (++) (potentially viable for days to months) or (+) (generally viable for minutes to hours). The ratings system for environmental stability was assigned to reflect the wide range of stability of the agents, while maintaining a simple overall scheme that contained only a few categories (minutes to hours, days to months, >1 year). The ratings for all the subcategories evaluated for production and dissemination potential were then totaled and agents were assigned a final rating for production and dissemination capability. If the total rating in the subcategories was ≥9, the agent was given (+++); for a total of 7-8, the agent was given a (++); and for a total of ≤6, the agent was given a final rating of (+) for the overall production and dissemination capability.

As potential outbreak propagation through continued person-to-person transmission would also increase the overall dissemination capabilities of an agent, they were evaluated separately for this characteristic. Agents were rated highest if they had potential for both person-to-person respiratory and contact spread (+++) and lower for mainly respiratory (++) or contact spread potential alone (+). Agents were rated (0) if they presented low or no transmission risk.

Agents were also assessed (0 to +++) according to preexisting heightened public awareness and interest, which may contribute to mass public fear or panic in biological terrorism events. The

number of times an agent or disease appeared in a selected form of media was used as a surrogate to determine the current level of public awareness and interest for the agent or disease. Titles of newspaper articles and radio and television transcripts from June 1, 1998, to June 1, 1999, in an Internet database (9) were retrospectively searched by agent name and disease. This database contained articles and transcripts from approximately 233 newspapers and 70 radio or television sources. If a disease was caused by multiple agents (e.g., viral hemorrhagic fever), the database was searched for each of the agents in addition to the name of the disease. Articles or transcripts were only counted if the name of the agent, disease, or other general terms such as bioterrorism, biological terrorism, terrorism, and weapons of mass destruction appeared in the title. Multiple hits for the same title were counted only once unless they appeared in different newspapers or transcripts. Agents were rated based on the number of times they appeared in these forms of media within the 1-year period. Agents were given (0) rating for <5 titles, (+) for 5-20 titles, (++) for 21-45 titles, and (+++) for >45 titles identified within the search period.

Requirements for special public health preparedness were also considered. Higher ratings were given to agents with different requirements for special preparedness. An agent was given a (+) for each special preparedness activity that would be required to enhance the public health response to that agent. These distinct preparedness requirements included 1) stockpiling of therapeutics to assure treatment of large numbers of people (+), 2) need for enhanced public health surveillance and education (+), and 3) augmentation of rapid laboratory diagnostic capabilities (+). Therefore, if all three special preparedness efforts would be required to provide a strong public health response for that agent, it was given (+++) for this category. Agents that did not require all special preparedness efforts were given lower ratings (++ or +).

References

1. Benenson AS, ed. Control of communicable diseases manual. 16th ed. Washington: American Public Health Association; 1995.

2. Pickering LK, ed. 2000 Red book: report of the Committee on Infectious Diseases. 25th ed. Elk Grove Village (IL): American Academy of Pediatrics; 2000.

3. Mandell GL, Bennett JE, Dolin R, eds. Principles and practices of infectious diseases. 5th ed. Philadelphia: Churchill Livingstone; 2000.

4. U.S. Department of Health and Human Services. Biosafety in microbiological and biomedical laboratories. 4th ed. Washington: U.S. Government Printing Office; 1999.

5. Eitzen E, Pavlin J, Cieslak T, Christopher G, Culpepper R, eds. Medical management of biological casualties handbook. 3rd ed. Frederick (MD): U.S. Army Medical Research Institute of Infectious Diseases; 1998.

6. Murray PR, Baron EJ, Pfaller MA, Tenover FC, Yolken RH, eds. Manual of clinical microbiology. 7th ed. Washington: ASM Press; 1999.

7. Fenner F, Henderson DA, Arita I, Jezek Z, Ladnyi ID. Smallpox and its eradication. Geneva: World Health Organization; 1988.

8. Dixon CW. Smallpox. London: Churchill; 1962.

9. Electronic Library Personal Edition at http://www.elibrary.com/.
